# Modeling Multiple Item Context Effects With Generalized Linear Mixed Models

**DOI:** 10.3389/fpsyg.2019.00248

**Published:** 2019-02-25

**Authors:** Norman Rose, Gabriel Nagy, Benjamin Nagengast, Andreas Frey, Michael Becker

**Affiliations:** ^1^Hector Research Institute of Education Sciences and Psychology, University of Tübingen, Tübingen, Germany; ^2^Leibniz Institute for Science and Mathematics Education, Kiel, Germany; ^3^Department of Educational Psychology, Measurement, Evaluation and Counseling, Institute of Psychology, Goethe-University Frankfurt, Frankfurt, Germany; ^4^Faculty of Education, Centre for Educational Measurement, University of Oslo, Oslo, Norway; ^5^German Institute for International Educational Research, Frankfurt, Germany

**Keywords:** item position effects, item context effects, domain order effects, multidimensional item response theory, generalized linear mixed models

## Abstract

Item context effects refer to the impact of features of a test on an examinee's item responses. These effects cannot be explained by the abilities measured by the test. Investigations typically focus on only a single type of item context effects, such as item position effects, or mode effects, thereby ignoring the fact that different item context effects might operate simultaneously. In this study, two different types of context effects were modeled simultaneously drawing on data from an item calibration study of a multidimensional computerized test (*N* = 1,632) assessing student competencies in mathematics, science, and reading. We present a generalized linear mixed model (GLMM) parameterization of the multidimensional Rasch model including *item position effects* (distinguishing between *within-block position effects* and *block position effects*), *domain order effects*, and the interactions between them. Results show that both types of context effects played a role, and that the moderating effect of domain orders was very strong. The findings have direct consequences for planning and applying mixed domain assessment designs.

## Introduction

Psychological tests including achievement test aim at inferring person's unobservable characteristics from their observed response behavior to a set of stimuli, e.g., test items. If different forms of a particular test exist, it is commonly assumed that the persons' response behavior to the items is independent of the choice of the test form. Violations of this assumption are referred to as item context effects, i.e., the test forms are an unintended source of variability in item and test scores. Ignoring this construct-irrelevant variance can lead to biased inference about person's characteristic measured by the test as well as item characteristics (e.g., item difficulty and item discrimination), and test characteristics (e.g., reliability and validity). Defining item context effects as systematic effects of the test form on the persons' response behavior suggests that potentially many different item context effects exist depending on the properties that differ between the test forms. Well-known item context effects are *item position effects, test mode effects* and *domain-order effects*.

*Item position effects* refer to systematic changes of item characteristics represented by item parameter estimates depending on its position within a test (Mollenkopf, 1950; Leary and Dorans, 1985; Yousfi and Böhme, 2012). In test for assessing multiple constructs or domains (e.g., multiple latent traits), the order in which the domains are measured by the items of the test can affect the persons' response behavior. This specific type of order effects are referred to as *domain order effects*. Sytematic differences in the persons' item responses to computerized tests compared to traditional paper-and-pecil tests are denoted by *test mode effects*.

Empirical findings suggest that item context effects are quite common. For example, *item position effects* on item difficulty appear to play a role in virtually all moderate to long achievement tests. Most empirical results indicate that items become increasingly difficult when put toward the end of a test (e.g., Hohensinn et al., 2008; Meyers et al., 2009; Hartig and Buchholz, 2012; Albano, 2013; Debeer and Janssen, 2013; Frey et al., 2017). *Domain order effects* are well-documented in the area of attitude measurement [e.g., Harrison and Mclaughlin (1993)], and also appear to play a role in achievement tests (Mazzeo and Von Davier, 2009). Similarly*, test mode effects* are a common area of research. A meta-analysis by Mead and Drasgow (1993) found no test mode effects for power tests but substantial test mode effects for speeded cognitive ability tests.

Most empirical studies focus on just one type of item context effects. However, different types of item context effects are likely to operate simultaneously and may interact with each other. The (position) effect of putting on an item toward the end of a test might depend on the kind of items presented beforehand. Hence, such effects appear to be exaggerated in booklet designs (Shoemaker, 1973; Frey et al., 2009) commonly employed in large-scale assessments (LSA) of student achievement. Many LSAs use booklet designs in which items appear in different positions within different booklets and are preceded and followed by different items from potentially different domains (e.g., reading and mathematics). An even more extreme case is multi-dimensional computerized adaptive testing (MCAT; Segall, 1996, 2000; Frey and Seitz, 2009). In contrast to booklet designs, the test form does not exist prior to the test in MCAT. Rather, the individually selected set of items depends on the test taker's ongoing response behavior in the assessment.

Item context effects violate the assumption of standard item response theory (IRT) models commonly employed for scoring items and persons. In particular, it is assumed that item and person parameters are invariant across test forms and are stable across the course of the testing session. Erroneously assuming the absence of position and domain order effects is likely to result in biased item and person parameter estimates and can, therefore, threaten the validity of test score interpretations and uses. For example, if mathematics items become more difficult when presented after science items compared to reading items in a multidimensional achievement test, the variation in the item difficulties needs to be taken into account when estimating the persons' mathematics competencies. Otherwise, spurious mean differences in the mathematics competencies result between the test takers depending on the test forms. Existing IRT models can be adopted to account for position and domain order effects as well as their interactions. Doing so enables researchers to assess and to statistically control for such effects and also allows for fair comparisons of test scores across test forms. The choice of models depends on the specific item context effects that are considered. If the number of test forms or test modes is small to moderate multiple group IRT models or multi-facet IRT models can be used. Explanatory IRT models based on generalized linear mixed models (GLMM) are very flexible in modeling multiple item context effects.

The aims of this article are 2-fold. First, we show how different types of item context effects can be analyzed simultaneously using generalized linear mixed models (GLMM; McCulloch et al., 2008; Stroup, 2012). Our proposed model builds upon a multidimensional Rasch model defined by content domains (mathematics, science, and reading) extended for the impact of item positions and domain orders, as well as their interactions. We exemplify how such a model can be theoretically derived step-by-step depending on the peculiarities of the item and test design. Second, we examine item position effects and domain order effects in a test consisting of typical test material employed in LSAs of student achievement. Therefore, the model can directly be used as a template in other studies with the same conceptual test design. The data stems from a large calibration study of three computerized adaptive tests assessing mathematics, science, and reading, employing a booklet design ideally suited for this purpose. This is, the first application considering both types of context effects simultaneously.

This paper is organized as follows. We start with a brief review of the theoretical aspects of context effects and clarify the terminology used in the later parts of the paper. Subsequently, we present a short overview of existing model-based methods for surveying item context effects, including the GLMM used in this study. In the method section, the study design, the sample, and the data will be introduced. Based on the booklet design presented, we then formulate a series of models with increasing complexity accounting for context effects. After presenting the results of the different models, we close with a discussion of our findings and the implications of our study.

## Theoretical Background

### Context Effects: Position and Domain Order Effects

Following Yousfi and Böhme (2012), we use the term *item context effects* as the generic term, with *item position effects* and *domain order effects* as special instances thereof. In this paper, we refer to item position effects as differences in the distribution of the item score *Y*_*j*_ of an item *j* depending on the position in which the item is presented in the test or the booklet. In multidimensional tests measuring multiple dimensions such as achievement domains, domain order effects may occur. Domain order effects refer to distributional differences in item scores of one or more items of a domain *d* depending on the order in which all domains are measured by the test. Item position effects and domain order effects may occur simultaneously and interact with each other. In particular, the size of item position effects may differ across domains, which means that the domain functions as a moderator of the item position effect. The domain order can also moderate item position effects, i.e., the item position effects in items of the same domain can vary depending on the domain that was previously presented.

Note that, in tests where items of the same domain are presented in succession, a block structure results, meaning that items belonging to the same domain are typically grouped together to form one block within the test. Hence, most mixed domain assessment designs, such as used in PISA (OECD, 2009, 2014) or TIMSS (Olson et al., 2008), might be considered as being composed of multiple blocks of items belonging to the same domain. In such tests individuals might first work on a block of items assessing, reading, then on a block of items assessing mathematics, and so on. Hence, in such a design it is useful to distinguish between the position of the block in which an item is presented and the position of the same item in the test. Although the block position and the item position are necessarily interdependent, separating these two factors facilitates analyses of multiple item context effects. For example, item position effects can be moderated by the block position. That is, a positive linear item position effect (practice effect) may occur when mathematic items are presented at the beginning of the test (Leary and Dorans, 1985; Nagy et al., 2018a), whereas a negative item position effect (fatigue effect) may occur if the same items are presented at the end of the test (Leary and Dorans, 1985; Ackerman and Kanter, 2009). In this article, we confine ourselves to investigating effects of the item position, the block position, and the domain order, as well as interactions between these factors. Note that, depending on the test design, additional item contexts could be identified (e.g., mode of presentation; computer-based vs. paper-based; Kröhne and Martens, 2011).

### Conceptualizing Item Context Effects

Context effects can be defined in a more formal way by considering the idea of conditional independence of item responses. The position of an item *j* in a test or the position of the block of domain *d*, as well as the order of domains in a test, are characteristics of a test. In order to generally define context effects, we can represent these factors as random variables *W*_*h*_. Let ***W*** = *W*_1_,…,*W*_*Z*_ be the vector of all potential item context factors. Furthermore, let ***Y*** = *Y*_1_,…,*Y*_*J*_ be the items constituting the measurement model *M*_0_ of a potentially multidimensional latent variable ***θ***_0_. Let **ϕ**_0_ the vector of model parameters of *M*_0_ including the item parameters. The subscript zero indicates that context effects are not considered in *M*_0_. Hence, item and person parameters are assumed to be invariant across test forms. The absence of item context effects can then be defined as the conditional stochastic independence

(1) $$\begin{array}{r} Y⊥W|\left(\theta_{0};\varphi_{0}\right) \end{array}$$

Item context effects exist if conditional independence—as expressed by Equation 1—does not hold. Whenever, the assumption of conditional independence is violated, context effects should be explicitly incorporated in the IRT model (Yousfi and Böhme, 2012). This leads to a model different from *M*_0_ as item and/or person parameters are allowed to be different across test forms depending on ***W***.

### Model-Based Approaches for Item Context Effects

Different approaches have been proposed for dealing with item context effects. Specific IRT models have been derived to account for a particular context effect. Especially item position effects received attention in educational LSA (Hohensinn et al., 2008, 2011; Hartig and Buchholz, 2012; Debeer and Janssen, 2013). Using terminology borrowed from multilevel modeling, existing models can be divided into two major classes: Fixed and random effects models (Bosker and Snijders, 2011). In fixed effects models, item context effects are represented by additional, invariant model parameters. For example, the Linear Logistic Test Model (LLTM; Fischer, 1973, 1995) has been adapted to analyze item position effects (Hohensinn et al., 2008, 2011).

In random effects models, item context effects can be represented as random variables. These models are of major importance if context effects are assumed to vary across items and/or persons. Wang and Wilson (2005) proposed the use of random effects facet models to account for local dependencies, as implied by context and position effects. Multidimensional IRT (MIRT) models with fixed and random item position effects have been proposed by Debeer and Janssen (2013).

Because of their flexibility, GLMMs (McCulloch et al., 2008; Stroup, 2012) recently also became popular in psychometrics. In this framework, item and/or person parameters are represented as random effects underlying the observed item responses. Explanatory IRT models are a class of GLMMs including additional covariates in modeling item responses (Kamata, 2001; Rijmen et al., 2003; De Boeck and Wilson, 2004; Janssen et al., 2004; Van den Noortgate and De Boeck, 2005). Accordingly, the GLMM framework can be used to model fixed and random item context effects by including context variables, such as the item position, as additional predictors in the model (Hartig and Buchholz, 2012; Debeer and Janssen, 2013). Many models specified in the GLMM framework can be translated into equivalent models in the MIRT framework, and vice versa. Due to its flexibility, the GLMM framework allows for modeling the impact of multiple context characteristics and their possible interactions.

## The Present Study

Our study focused on item position and domain order effects on subjects' item responses in a mixed domain design consisting of test material typically employed in recent LSAs of student achievement. In this study, we used data from a study whose test design allows assessing both kinds of context effects and their interactions. Our main question was whether item position effects were moderated by the order of domains measured in the different test booklets. From a substantive perspective our results are informative for researchers planning assessments in which multiple domains are assessed. The existence of domain order effects operating in addition to position effects might be an important issue to be considered in large scale studies of student achievement. Typical assessments with different test forms seek to control potential position effects by design (i.e., balancing; Frey et al., 2009). However, when domain order effects exist in addition to position effects the commonly used designs may fall short in achieving this goal. Additionally, domain orders are often perfectly confounded with item (block) positions, meaning that position and domain order effects cannot be separated unambiguously. For example, the PISA data-base has been used to study item position effects (e.g., Hartig and Buchholz, 2012; Debeer and Janssen, 2013; Nagy et al., 2018a), although position and domain order effects cannot be thoroughly separated from each other.

A second goal of this article is to exemplify the use of GLMMs for the simultaneous analysis of item position and domain order effects. We will present a sequence of multidimensional IRT models specified in the GLMM framework. A step by step derivation of the models will be provided taking the assessment design into account. As we will show in the remainder of the article the GLMM framework provides great flexibility for modeling the impact of features of the assessment design on individuals' item responses that are not easily implemented in the classical MIRT framework.

### Methods

#### Sample

The data set consisted of 49,128 responses gathered in a calibration study for three tests measuring student achievement in the domains of mathematics, science, and reading within a research project (for more information see Ziegler et al., 2016; Spoden et al., 2018). The study was carried out in accordance with the recommendations of the German Research Foundation (DFG) with written informed consent from all subjects. The fulfillment of these recommendations was approved by the three German Federal States of Thuringia, Lower Saxony, and Hessia, in which data were collected. All subjects participated voluntarily and gave written informed consent in accordance with the Declaration of Helsinki. The sample consisted of *N* = 1,632 students (46% female) being in vocational education and training in Germany which is organized. The majority of test takers (66%) were in their third year of vocational training. The mean age was *M* = 21.36 (*SD* = 3.03). Eighty-Seven Percent of the test takers had German citizenship. Since the analysis of potential domain order effects is central in our study, we only included test takers who answered items from at least two domains and discarded those who provided item responses in only one of the three domains. Therefore, we used 48,986 responses from *N* = 1,598 test takers in the further statistical analyses. Test takers completed, on average, 31 items (25th quantile = 31, 50th quantile, and 75th quantile = 33).

#### Booklet Design and Assessment Procedure

Achievement was assessed by an item pool consisting of 339 items (133 mathematics, 133 science, and 73 reading items). The distribution of the items to the test takers was accomplished with a two-level booklet design. At the first level, domain-specific blocks of items were balanced (Table 1). The blocks for mathematics, science, and reading contained 12, 12, and 9 items respectively. In each of the 18 cells of level one, a Youden Square Design (YSD) was constructed using the freely available software Youden (Frey and Annageldyev, 2015). YSDs are balanced incomplete block designs that are frequently used as booklet designs in LSAs such as PISA. The YSD used in this study assured that all items appeared with equal frequency in the complete set of test booklets, that each item appeared in each position in a test booklet with equal frequency, and that each pair of items appeared together in a test booklet with equal frequency.

**Table 1:** The six domain orders used in the test booklets (Youden squares).

	**Booklet**					
**Block position**	**1**	**2**	**3**	**4**	**5**	**6**
1	Reading	Reading	Science	Science	Mathematics	Mathematics
2	Mathematics	Science	Reading	Mathematics	Science	Reading
3	Science	Mathematics	Mathematics	Reading	Reading	Science

The booklet design implied that each mathematics and science item was presented in the positions 1 to 33. As only 9 reading items were included in a booklet, reading items were presented in the positions 1–9, 13–21, and 25–33. Between 123 and 199 responses were observed for each item (mathematics: *M* = 133.39, range = 125–143; science: *M* = 133.69, range = 123–144; reading: *M* = 188.10, range = 168–199). On average, 4.04 responses were observed for each mathematics items in each position. 4.05 responses to each science item and 6.97 responses to each reading item were, on average, obtained in each position.

The tests were administered as an online computer-based test. Each testing session started with a 10-min-long standardized instruction. The test takers were informed about the domains being assessed, but participants did not know the order of the domains in their individually assigned test form. The test forms were randomly assigned to the students, who had, in total, 60 min to complete the test.

#### IRT Models for Item Position and Domain Order Effects

The data were analyzed by a series of GLMMs, assuming that both persons and items were random (De Boeck, 2008). Items were considered as random because of the large number of items available (339 items), making the estimation of separate item parameters impractical. Considering items and persons to be random results in a crossed-random structure (Locker et al., 2007; Baayen et al., 2008). The responses are nested within items and persons. The person side was modeled by three correlated random effects referring to the domain specific ability variables mathematics (θ_*M*_), science (θ_*S*_), and reading (θ_*R*_). The ability vector ***θ*** was assumed to follow a multivariate normal distribution MVN(0, **Σ**) with the mean *E*(***θ***) = **0** and the unrestricted covariance matrix **Σ**. On the item side, we assumed three independent random effects ζ_*M*_, ζ_*S*_, and ζ_*R*_, which were assumed to be normally distributed with zero mean and *Var*(ζ_*d*_) each.

In developing the full model, we began with M0, the three-dimensional random Rasch model (De Boeck, 2008), not accounting for context effects. This model can be written as a random slope model for the indicator variables *I*_*M*_, *I*_*S*_, and *I*_*R*_, which indicate whether the response *Y*_*ijd*_ refers to a mathematics, science, or reading item. The level-1 model equation of the logit of person *i* who answered item *j* of domain *d* can be written as

(2) $$\begin{array}{r} l\left(Y_{ijd}\right)=\beta_{ijM}I_{M}+\beta_{ijS}I_{S}+\beta_{ijR}I_{R} \end{array}$$

The level-2 model equation of the random slope of domain *d* is

(3) $$\begin{array}{r} \beta_{ijd}=\gamma_{d}+\theta_{id}+\zeta_{jd} \end{array}$$

where γ_*d*_ is the fixed effect for domain *d*. As *E*(***θ***) = **0**, γ_*d*_ can be interpreted as the mean domain-specific item easiness.

Item position effects are the most frequently investigated type of item context effects. Such effects are incorporated into model M1. We entered the position *p* of item *j* of domain *d* presented to person *i* as a covariate denoted by *X*_*ijpd*_. To simplify the notification, we simply write *X*_*p*_ in the remainder. *X*_*p*_ is the level-1 covariate, so that Equation 1 extends to

(4) $$\begin{array}{r} l\left(Y_{ijpd}\right)=\beta_{ijM}I_{M}+\beta_{ijS}I_{S}+\beta_{ijR}I_{R}+\lambda_{d}X_{p} \end{array}$$

To facilitate the interpretation of model parameters, *X*_*p*_might be standardized. In our case, we set *X*_*p*_ = (*p*−17)/32. As a result, the coefficient λ_*d*_ was the expected change in the logit of a randomly drawn person solving a randomly selected item of domain *d* when this item is presented at position 33 instead of the first position in the test. In the model suggested, we assumed the existence of domain specific item position effects by allowing the regression coefficient λ_*d*_ to vary across the domains, so that

(5) $$\begin{array}{r} \lambda_{d}=\kappa_{M}I_{M}+\kappa_{S}I_{S}+\kappa_{R}I_{R} \end{array}$$

In Equation 5, κ_*d*_ is the mean logit change in a randomly selected item out of domain *d* if it were presented in the last instead of the first position of the test.

The model M1 is an explanatory IRT model with the item position as a level-1 predictor. The main restriction of this model is that the item position effect has a linear form, and that the item position effect is a fixed effect which does not have different values for different items and/or persons. Both restrictions can be relaxed in the GLMM framework. Nonlinear forms of item position effects can be examined by adding higher-order polynomials of the position variable *X*_*p*_(e.g., $X_{p}^{2}$ and $X_{p}^{3}$). Random item position effects across persons and across items can be taken into account by defining a random coefficient λ_*ijd*_ that can vary across persons and items. In the present study we also employed these extended parameterizations of M1 by checking for nonlinear trends, and for random effects on the person and item side. However, as we found no evidence for random effects, we do not investigate this issue any further.

As M1 only accounts for item position effects, the model was extended to include domain order effects, leading to a new model (M2). M2 not only assumed position and domain order effects, but also allowed for interactions between the two effects. For example, a position effect in science items may be stronger or weaker depending on whether mathematics or reading items were assigned previously. Following this idea, we took the domain order (as an additional predictor) into account in M2.

In the booklet design employed in this article, item position and domain order are not independent from one another. If, for example, a mathematics item was presented in a test booklet of the domain order mathematics (*M*), science (*S*), and reading (*R*), then the item was necessarily presented in one of the positions 1–12. Hence, conditioning on the domain order restricts the range of possible positions in which items of a particular domain can be presented. Therefore, the block structure of the test needs to be taken into account.

We included variables *B*_*ijdb*_ which indicate that an item *j* of domain *d* was presented to person *i* in block position *b* of the test. To keep notation simple, we use the short form *B*_*b*_ in the remainder of the article. The first item block position served as the reference block, so that two additional indicator variables *B*_2_ and *B*_3_ were included that jointly indicate a response to an item of the first block (*B*_2_ = 0, *B*_3_ = 0), the second block (*B*_2_ = 1, *B*_3_ = 0), or the third block (*B*_2_ = 0, *B*_3_ = 1).

In order to yield a model with parameters that can be interpreted unequivocally, the item positions' were within-block standardized in M2 as *X*_*pb*_ = (2*p*_*b*_−*P*_*b*_−1)/2(*P*_*b*_−1), where *P*_*b*_ stands for number of items in block *b*, and *p*_*b*_ refers to the within-block item position of item *j*. That is, the variable *X*_*pb*_ always has a value of −0.5 when an item *j* was presented in the first position of block *b*, whereas a value of 0.5 indicates that the item was presented at the last position of block *b*. Due to the within-block standardization of the item position variable, the logistic regression coefficients attached to *X*_*pb*_ stand for the logit change if a randomly chosen item in domain *d* is presented in the last instead of in the first position of block *b*.

So far, the model equation of model M2 can be written as:

(6) $$\begin{array}{l} l\left(Y_{ijdpbt}\right)=\beta_{ijM}I_{M}+\beta_{ijS}I_{S}+\beta_{ijS}I_{S}+\alpha_{d2t}B_{2}+\alpha_{d3t}B_{3}\\ \;+\lambda_{dbt}X_{pb}, \end{array}$$

where α_*d*2*t*_ and α_*d*3*t*_ stand for the effects of the block positions indicated by *B*_2_ and *B*_3_, and λ_*dbt*_ stands for the within-block position effect. Note that these parameters are indexed by a newly introduced index *t* standing for the domain order *T*. Hence, block position effects can have different values depending on the domain *d* and the domain order *t*, whereas the size of the within-block item position effects might also depend on the block *b* besides the domain *d*, and the domain order *T*.

Formally, α_*dbt*_ is defined as a function *f*_(*D, T*)_ of the domain *D* = *d* and the domain order of a test booklet, denoted by *T* = *t*. Hence, the effect of a block *b* is allowed to have different values for each combination of *d* and *t* (e.g., the effect of *b* = 2 might be weakest in the science domain when assessed after a reading block). Similarly, λ_*dbt*_ is defined as a function *f*_(D, *B, T*)_ of the domain, the block position and the domain order. So, the item position effects may vary depending on the domain *D* = *d*, the position of the block *B* = *b*, and the domain order *T* = *t*.

In the present investigation, the variable *T* has six values, referring to the six possible orders of the three domains; mathematics (*M*), science (*S*), and reading (*R*). In our final model, we used six indictor variables *T*_*MSR*_, *T*_*MRS*_, *T*_*SMR*_, *T*_*SRM*_, *T*_*RMS*_, and *T*_*RSM*_. The order of subscripts represents the domain order in a test booklet. It is important to note that, in the present case, each combination of a domain *d* and a block position *b* is only consistent with two domain orders. If, for example, science was presented in the second item block, it was necessarily administered in a test with either the domain order *M*-*S*-*R* or *R*-*S*-*M*. This means that a block position effect and a within-block item position effect in a given domain and block (i.e., a specific combination of *d* and *b*) can only have two different values that depend on the possible domain orders. Furthermore, as it appears reasonable that individuals are not affected by forthcoming parts of the test, the impact of domain order effects could be constrained for the first block position *b* = 1. For example, the within-block item position effects in mathematics assessed in the first item block position should not be affected by the domains assessed in the subsequent block.

Given the aforementioned restrictions on the impact of domain orders, and assuming the existence of linear item position effects within item blocks, the full reduced-form model equation of M2 recurring on domain order indicators is given as:

(7) $$\begin{array}{l} l\left(Y_{ijdpb}\right)=\gamma_{M1}I_{M}+\gamma_{S1}I_{S}+\gamma_{R1}I_{R}\\ \;+\gamma_{M2}^{\left(S\right)}I_{M}B_{2}+\gamma_{M2}^{\left(\times \right)}T_{RMS}I_{M}B_{2}+\gamma_{S2}^{\left(M\right)}I_{S}B_{2}\\ \;+\gamma_{S2}^{\left(\times \right)}T_{RSM}I_{S}B_{2}+\gamma_{R2}^{\left(M\right)}I_{R}B_{2}+\gamma_{R2}^{\left(\times \right)}T_{SRM}I_{R}B_{2}\;\\ \;+\gamma_{M3}^{\left(SR\right)}I_{M}B_{3}+\gamma_{M3}^{\left(\times \right)}T_{RSM}I_{M}B_{3}+\gamma_{S3}^{\left(MR\right)}I_{S}B_{3}\;\\ \;+\gamma_{S3}^{\left(\times \right)}T_{RMS}I_{S}B_{3}+\gamma_{R3}^{\left(MS\right)}I_{R}B_{3}+\gamma_{R3}^{\left(\times \right)}T_{SMR}I_{R}B_{3}\\ \;+\kappa_{M1}B_{1}I_{M}X_{pb}+\kappa_{S1}B_{1}I_{S}X_{pb}+\kappa_{R1}B_{1}I_{R}X_{pb}\\ \;+\kappa_{M2}^{\left(S\right)}B_{2}I_{M}X_{pb}+\kappa_{M2}^{\left(\times \right)}T_{RMS}B_{2}I_{M}X_{pb}\\ \;+\kappa_{M3}^{\left(SR\right)}B_{3}I_{M}X_{pb}+\kappa_{M3}^{\left(\times \right)}T_{RSM}B_{3}I_{M}X_{pb}\\ \;+\kappa_{S2}^{\left(M\right)}B_{2}I_{S}X_{pb}+\kappa_{S2}^{\left(\times \right)}T_{RSM}B_{2}I_{S}X_{pb}+\kappa_{S3}^{\left(MR\right)}B_{3}I_{S}X_{pb}\\ \;+\kappa_{S3}^{\left(\times \right)}T_{RMS}B_{3}I_{S}X_{pb}\\ \;+\kappa_{R2}^{\left(M\right)}B_{2}I_{R}X_{pb}+\kappa_{R2}^{\left(\times \right)}T_{SRM}B_{2}I_{R}X_{pb}+\kappa_{R3}^{\left(MS\right)}I_{R}X_{pb}\\ \;+\kappa_{R3}^{\left(\times \right)}T_{SMR}B_{3}I_{R}X_{pb}\\ \;+\theta_{iM}I_{M}+\zeta_{jM}I_{M}+\theta_{iS}I_{S}+\zeta_{jS}I_{S}+\theta_{iR}I_{R}+\zeta_{jR}I_{R} \end{array}$$

The stepwise derivation of this model is presented in Appendix A. Here, it should be noted that all effects of the item block position are represented by the parameters γ_*db*_, where the first subscript *d* indicates the domain of the item, and the second subscript *b* indicates the block in which the item was presented. The superscripts indicate the domains of items that were presented previously and the order of the domains. For example, $\gamma_{S2}^{\left(M\right)}$ indicates the average effect of presenting science items in block two (*b* = 2), following mathematics items presented in the first block (*b* = 1). Similarly, $\gamma_{M3}^{\left(SR\right)}$ is the average logit change when mathematics items are presented in the third item block of a test with the domain order *S-R-M* instead of the first item block. Differences in block position effects and within-block item position effects are marked by “^×^”. For example, only two possible domain orders—*M*-*S-R* and *R-S-M*—exist when science items are presented in the second item block. We chose the domain order *M*-*S-R* as the reference for block position effects in the science items of block two. Therefore, $\gamma_{S2}^{\left(\times \right)}$ is the difference $\gamma_{S2}^{\left(R\right)}-\gamma_{S2}^{\left(M\right)}$ and represents the interaction effect between domain order and block position within items of the same domain.

The domain-specific within-block item position effects in the item blocks one, two and three refer to the parameters κ_*d*1_, $\kappa_{d2}^{\left(.\right)}$, and $\kappa_{d3}^{\left(..\right)}$. The superscripts of $\kappa_{d2}^{\left(.\right)}$ and $\kappa_{d3}^{\left(..\right)}$ indicate the domains of previously presented items and their order. Differences between within-block item position effects in items of the same domain presented in the same item block are marked by (^×^). For example, $\kappa_{M2}^{\left(S\right)}$ is the average logit change when a randomly chosen mathematics item is presented in the last position of item block two in a test that started with science items. However, $\kappa_{M2}^{\left(\times \right)}$ is the difference $\kappa_{M2}^{\left(R\right)}-\kappa_{M2}^{\left(S\right)}$ between the item position effect of mathematics items of block two in a test with the domain order *R-M-S* instead of *S-M-R*. Hence, parameters $\kappa_{db}^{\left(\times \right)}$ represent the interaction of the domain, the block position, the domain order, and the within-block item position. The translation of the γ- and κ-parameters of Equation 7 into the α- and λ-parameters of Equation 6 is summarized in Table 2.

**Table 2:** Decomposition of block position effects α_dbt_ and within-block item position effects λ_dbt_ as a function of domain order specific effects (Equation 6).

**Block position *b* = 1**	**Block position *b* = 2**	**Block position *b* = 3**
**BLOCK POSITION EFFECTS α*_dbt_***		
	$\alpha_{M2\left(SMR\right)}=\gamma_{M2}^{\left(S\right)}$	$\alpha_{M3\left(SRM\right)}=\gamma_{M3}^{\left(SR\right)}$
	$\alpha_{M2\left(RMS\right)}=\gamma_{M2}^{\left(S\right)}+\gamma_{M2}^{\left(\times \right)}$	$\alpha_{M3\left(RSM\right)}=\gamma_{M3}^{\left(SR\right)}+\gamma_{M3}^{\left(\times \right)}$
	$\alpha_{S2\left(MSR\right)}=\gamma_{S2}^{\left(M\right)}$	$\alpha_{S3\left(MRS\right)}=\gamma_{S3}^{\left(MR\right)}$
	$\alpha_{S2\left(RSM\right)}=\gamma_{S2}^{\left(M\right)}+\gamma_{S2}^{\left(\times \right)}$	$\alpha_{S3\left(RMS\right)}=\gamma_{S3}^{\left(MR\right)}+\gamma_{S3}^{\left(\times \right)}$
	$\alpha_{R2\left(MRS\right)}=\gamma_{R2}^{\left(M\right)}$	$\alpha_{R3\left(MSR\right)}=\gamma_{R3}^{\left(MS\right)}$
	$\alpha_{R2\left(SRM\right)}=\gamma_{R2}^{\left(M\right)}+\gamma_{R2}^{\left(\times \right)}$	$\alpha_{R3\left(SMR\right)}=\gamma_{R3}^{\left(MS\right)}+\gamma_{R3}^{\left(\times \right)}$
**WITHIN-BLOCK ITEM POSITION EFFECTS λ_*dbt*_**		
λ_*M*1(*MSR*)_ = κ_*M*1_	$\lambda_{M2\left(SMR\right)}=\kappa_{M2}^{\left(S\right)}$	$\lambda_{M3\left(SRM\right)}=\kappa_{M3}^{\left(SR\right)}$
λ_*M*1(*MRS*)_ = κ_*M*1_	$\lambda_{M2\left(RMS\right)}=\kappa_{M2}^{\left(S\right)}+\kappa_{M2}^{\left(\times \right)}$	$\lambda_{M3\left(RSM\right)}=\kappa_{M3}^{\left(SR\right)}+\kappa_{M3}^{\left(\times \right)}$
λ_*S*1(*SMR*)_ = κ_*S*1_	$\lambda_{S2\left(MSR\right)}=\kappa_{S2}^{\left(M\right)}$	$\lambda_{S3\left(MRS\right)}=\kappa_{S3}^{\left(MR\right)}$
λ_*S*1(*SRM*)_ = κ_*S*1_	$\lambda_{S2\left(RSM\right)}=\kappa_{S2}^{\left(M\right)}+\kappa_{S2}^{\left(\times \right)}$	$\lambda_{S3\left(RMS\right)}=\kappa_{S3}^{\left(MR\right)}+\kappa_{S3}^{\left(\times \right)}$
λ_*R*1(*RMS*)_ = κ_*R*1_	$\lambda_{R2\left(MRS\right)}=\kappa_{R2}^{\left(M\right)}$	$\lambda_{R3\left(MSR\right)}=\kappa_{R3}^{\left(MS\right)}$
λ_*R*1(*RSM*)_ = κ_*R*1_	$\lambda_{R2\left(SRM\right)}=\kappa_{R2}^{\left(M\right)}+\kappa_{R2}^{\left(\times \right)}$	$\lambda_{R3\left(SMR\right)}=\kappa_{R3}^{\left(MS\right)}+\kappa_{R3}^{\left(\times \right)}$

*Block position effects for b = 1 are not available because b = 1 serves as the reference position for defining block position effects for b > 1*.

The most complex model (M2) presented here was developed based on the booklet design employed in the present study (Table 1). However, the setup provided here can be accommodated to fit other booklet specifications, thereby providing a general model for studying within-block position effects, block position effects, domain order effects, and their interactions.

#### Model Estimation and Hypothesis Testing

All models presented here can be fitted in *R* using the *glmer*-function of the lme4-package (Bates et al., 2014) with ML estimation, thereby allowing model comparisons by the likelihood ratio (LR) test. We also used the Akaike's information criterion (AIC) and the Bayesian information criterion (BIC) for model comparison. Additional hypotheses, which could be formulated as linear and non-linear functions of parameters of fitted models, were tested by means of the Delta method (Oehlert, 1992). In our analyses we used the *deltamethod* function implemented in the R-package car (Fox and Weisberg, 2011).

### Results

#### Model Comparisons

In this section, we present the results of the models M0, M1, and M2 that were used to test increasingly complex hypotheses about item position and domain order effects. The results are presented for each model separately, starting with the three-dimensional Rasch model with random item- and person-effects (M0). No context effects were taken into account in M0, which mainly serves for comparison. The standard deviations of the three latent person variables ranged from 0.710 in science to 0.867 in mathematics (Table 3). The latent abilities were strongly correlated with each other (0.832–0.907; see Table 3). The estimated standard deviations of the three random item effects ranged from 1.075 in reading to 1.316 in mathematics. The coefficients ${\hat{\gamma }}_{M}$ = 0.098 (*SE* = 0.117, *p* = 0.408), ${\hat{\gamma }}_{S}$ = 0.503 (*SE* = 0.114, *p* < 0.001), and ${\hat{\gamma }}_{R}$ = 0.334 (*SE* = 0.129, *p* = 0.010) can be interpreted as differences between the means of person's latent proficiency variables θ_*d*_ and the item difficulties of items belonging to domain *d*. Thus, the mean trait level in science was 0.503 logits higher than the mean item difficulty of science items. The same holds true for reading items. No mean difference was found between trait and item difficulties in mathematics.

**Table 3:** Estimated standard deviations and correlations of random effects of the different models.

**Model**	**Domain**	**Items**	**Persons**		
		***SD(ζdj)***	***SD(θdi)***	**Correlations**	
				**Mathematics**	**Science**
M0	Mathematics	1.316	0.866		
	Science	1.268	0.710	0.907	
	Reading	1.075	0.799	0.832	0.839
M1	Mathematics	1.316	0.859		
	Science	1.269	0.697	0.909	
	Reading	1.077	0.799	0.847	0.854
M2r	Mathematics	1.315	0.836		
	Science	1.268	0.680	0.913	
	Reading	1.077	0.770	0.848	0.854

Model M1 allows for estimating and testing the interaction between item position and the domain to study differences in position effects across the three domains mathematics, science, and reading. Before we fitted the multidimensional model M1 to the data, we first applied unidimensional models separately to each domain in order to find the functional form best suited for describing the item position effects. Possible nonlinear position effects were explored by including quadratic and cubic terms into the models. Based on LR tests, models with linear position effects were preferred for science [χ^2^(2) = 2.806, *p* = 0.246] and reading [χ^2^(2) = 2.142, *p* = 0.343]. For mathematics, a model including a nonlinear position effect was superior in terms of model fit [χ^2^(2) = 12.560, *p* = 0.002]. Linear position effects found for science (${\hat{\kappa }}_{S}$ = −0.487, *p* < 0.001) and reading (${\hat{\kappa }}_{R}$ = −0.224, *p* = 0.009) were negative. Hence, logits decreased on average by 0.487 (science) and 0.224 (reading), when an item's position in the test changed from the first to the last position in the test. In mathematics, only the coefficient of the quadratic item position term was significantly negative (${\hat{\kappa }}_{M}\left(qu.\right)$ = −0.986, *p* < 0.001), whereas coefficients of the linear and the cubic term did not differ significantly from zero [linear: ${\hat{\kappa }}_{M}\left(lin.\right)$ = 0.056, *p* = 0.745; cubic: ${\hat{\kappa }}_{M}\left(cub.\right)$ = −0.351, *p* = 0.692]. Hence, correct responses to mathematics items were, on average, more likely when presented in the middle instead of the beginning or the end of the test.

**Figure 1:**
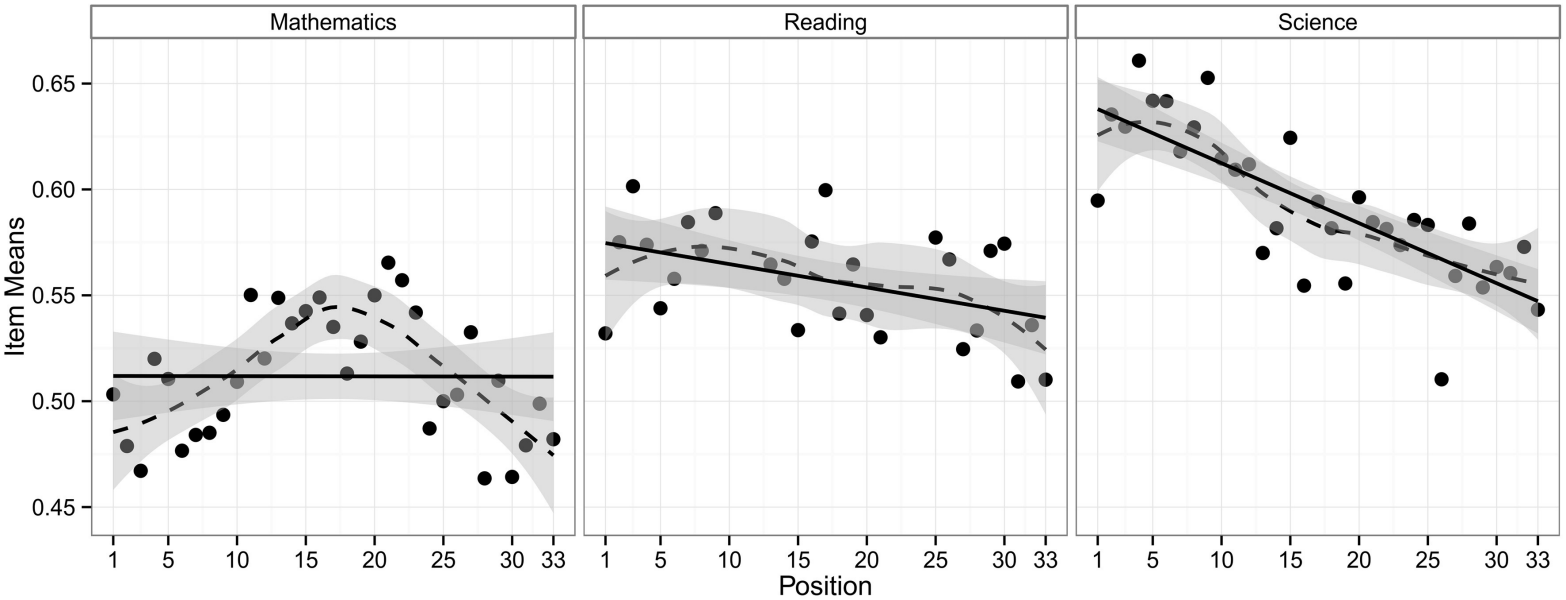
Proportions of correct responses averaged over all items of a domain depending on the item position within the test.

The multidimensional model M1 thus included linear item position effects for reading and science that were allowed to be different in magnitude, and a quadratic item position effect for mathematics. An LR test was applied to test the domain-by-item position interaction effect, providing a statistically significant interaction effect [χ^2^(2) = 12.650, *p* = 0.002]. This interaction effect is illustrated in Figure 1, which shows the observed mean proportions of correct responses averaged over all items, depending on the item position and the domain. We did not extend M1 for random item position effects across persons and items, because our data did not support their existence^1^.

In M2, the item position was broken down into the block position in which items of the same domain were administered in the test and the within-block item position. Table 4 shows the estimates of model M2. The resulting pattern revealed significant block position and block position-by-domain order interaction effects (see Table 4, upper three panels, *fixed effects referring to block position 1, 2 or 3*). With the exception of a single parameter estimate, all within-block item position effects and corresponding interaction effects were not significantly different from zero (see Table 4, lower panel, *fixed effects of within-block item position effects*). Indeed, a reduced version of model M2 (i.e., M2r) without within-block position effects did not result in a significantly worsened model fit [χ^2^(15) = 18.097, *p* = 0.258]. Model M2r include only the block-position, the domain order and the interaction of these two item context factors. However, this model fitted significantly better to the data than model M1 [χ^2^(9) = 94.186, *p* < 0.001]. We also found that ignoring the interaction between the block-position and the domain order significantly worsened model fit [χ^2^(6) = 88.174, *p* < 0.001].

**Table 4:** Estimated fixed effects of Models M2 and M2r.

**Term**	**Param**.	**Estimate (*SE*)**					
		**Model M2**			**Model M2r**		
		**Est**	**SE**	***p***	**Est**	**SE**	***p***
**FIXED EFFECTS REFERRING TO BLOCK POSITION 1**							
*I_*M*_*	γ_*M*_	−0.018	0.125	0.882	−0.019	0.125	0.877
*I_*S*_*	γ_*S*_	0.752^***^	0.120	< 0.001	0.751^***^	0.120	< 0.001
*I_*R*_*	γ_*R*_	0.402^**^	0.133	0.003	0.401^**^	0.133	0.003
**FIXED EFFECTS REFERRING TO BLOCK POSITION 2**							
*I_*M*_* × *B*_2_	$\gamma_{M2}^{\left(S\right)}$	0.415^***^	0.085	< 0.001	0.415^***^	0.085	< 0.001
*I_*S*_* × *B*_2_	$\gamma_{S2}^{\left(M\right)}$	−0.197^**^	0.076	0.010	−0.196^*^	0.076	0.003
*I_*R*_* × *B*_2_	$\gamma_{R2}^{\left(M\right)}$	−0.429^***^	0.078	< 0.001	−0.428^***^	0.078	< 0.001
*I_*M*_* × *B*_2_ × *T_*RMS*_*	$\gamma_{M2}^{\left(\times \right)}$	−0.268^**^	0.089	0.002	−0.265^**^	0.088	< 0.001
*I_*S*_* × *B*_2_ × *T_*RSM*_*	$\gamma_{S2}^{\left(\times \right)}$	−0.166^*^	0.082	0.042	−0.165^*^	0.082	0.010
*I_*R*_* × *B*_2_ × *T_*SRM*_*	$\gamma_{R2}^{\left(\times \right)}$	0.723^***^	0.094	< 0.001	0.723^***^	0.094	0.044
**FIXED EFFECTS REFERRING TO BLOCK POSITION 3**							
*I_*M*_* × *B*_3_	$\gamma_{M3}^{\left(SR\right)}$	0.338^***^	0.084	< 0.001	0.344^***^	0.084	< 0.001
*I_*S*_* × *B*_3_	$\gamma_{S3}^{\left(MR\right)}$	−0.564^***^	0.074	< 0.001	−0.561^***^	0.074	0.002
*I_*R*_* × *B*_3_	$\gamma_{R3}^{\left(MS\right)}$	−0.249^**^	0.082	0.002	−0.246^**^	0.082	< 0.001
*I_*M*_* × *B*_3_ × *T_*RSM*_*	$\gamma_{M3}^{\left(\times \right)}$	−0.518^***^	0.092	< 0.001	−0.521^***^	0.092	< 0.001
*I_*S*_* × *B*_3_ × *T_*RMS*_*	$\gamma_{S3}^{\left(\times \right)}$	0.246^**^	0.079	0.002	0.246^**^	0.079	0.003
*I_*R*_* × *B*_3_ × *T_*SMR*_*	$\gamma_{R3}^{\left(\times \right)}$	0.132	0.102	0.197	0.131	0.102	0.201
**FIXED EFFECTS OF WITHIN-BLOCK ITEM POSITION EFFECTS**							
*I_*M*_* × *B*_1_ × *X_*p*_*	κ_*M*1_	0.097	0.099	0.329			
*I_*S*_* × *B*_1_ × *X_*p*_*	κ_*S*1_	0.083	0.104	0.426			
*I_*R*_* × *B*_1_ × *X_*p*_*	κ_*R*1_	0.075	0.093	0.421			
*I_*M*_* × *B*_2_ × *X_*p*_*	$\kappa_{M2}^{\left(S\right)}$	0.232	0.15	0.121			
*I_*S*_* × *B*_2_ × *X_*p*_*	$\kappa_{S2}^{\left(M\right)}$	0.023	0.149	0.876			
*I_*R*_* × *B*_2_ × *X_*pb*_*	$\kappa_{R2}^{\left(M\right)}$	−0.009	0.15	0.952			
*I_*M*_* × *B*_2_ × *T_*RMS*_* × *X_*pb*_*	$\kappa_{M2}^{\left(\times \right)}$	−0.391^*^	0.191	0.041			
*I_*S*_* × *B*_2_ × *T_*RSM*_* × *X_*pb*_*	$\kappa_{S2}^{\left(\times \right)}$	−0.242	0.198	0.220			
*I_*R*_* × *B*_2_ × *T_*SRM*_* × *X_*pb*_*	$\kappa_{R2}^{\left(\times \right)}$	−0.225	0.217	0.298			
*I_*M*_* × *B*_3_ × *X_*pb*_*	$\kappa_{M3}^{\left(SR\right)}$	−0.209	0.152	0.168			
*I_*S*_* × *B*_3_ × *X_*pb*_*	$\kappa_{S3}^{\left(MR\right)}$	−0.149	0.144	0.303			
*I_*R*_* × *B*_3_ × *X_*pb*_*	$\kappa_{R3}^{\left(MS\right)}$	−0.294	0.165	0.075			
*I_*M*_* × *B*_3_ × *T_*RSM*_* × *X_*pb*_*	$\kappa_{M3}^{\left(\times \right)}$	0.107	0.205	0.602			
*I_*S*_* × *B*_3_ × *T_*RMS*_* × *X_*pb*_*	$\kappa_{S3}^{\left(\times \right)}$	0.076	0.195	0.697			
*I_*R*_* × *B*_3_ × *T_*SMR*_* × *X_*pb*_*	$\kappa_{R3}^{\left(\times \right)}$	0.199	0.242	0.410			

* *p < 0.05*,

** *p < 0.01*,

*** *p < 0.001*.

Table 5 shows the AIC and BIC values of the fitted models. The Model M0 has the highest and Model M2r the lowest AIC. Due to stronger penalty of model complexity the highest BIC value was found for Model M2. Model M1 shows the lowest BIC values. Nevertheless, we finally accepted M2r as the appropriate model to account for item context effects in our data, because of the substantial logit differences within single domains depending on the domain order. These results contradict the assumption of simple linear item position effects in Model M1. The final model M2r reveals that the response behavior is affected by the block position, the domain order and the interaction effects of both context factors, and that the items' absolute positions in the test have a rather small effect that appears to be negligible.

**Table 5:** AIC and BIC of the different models.

	**M0**	**M1**	**M2**	**M2r**
AIC	55585.62	55539.15	55474.87	55462.97
BIC	55691.21	55671.14	55818.04	55674.15

As parameters of logistic regressions with different fixed parts cannot be compared across models (Mood, 2010), we present the estimates of the fixed effects of both models, M2 and M2r in Table 4. The estimated standard deviations and correlations of the random effects of M0, M1, and M2r are presented in Table 3^2^. Due its complexity, we examine the results of Model M2r in more detail.

### Examination of Position Effects Moderated by Domain Order

**Figure 2:**
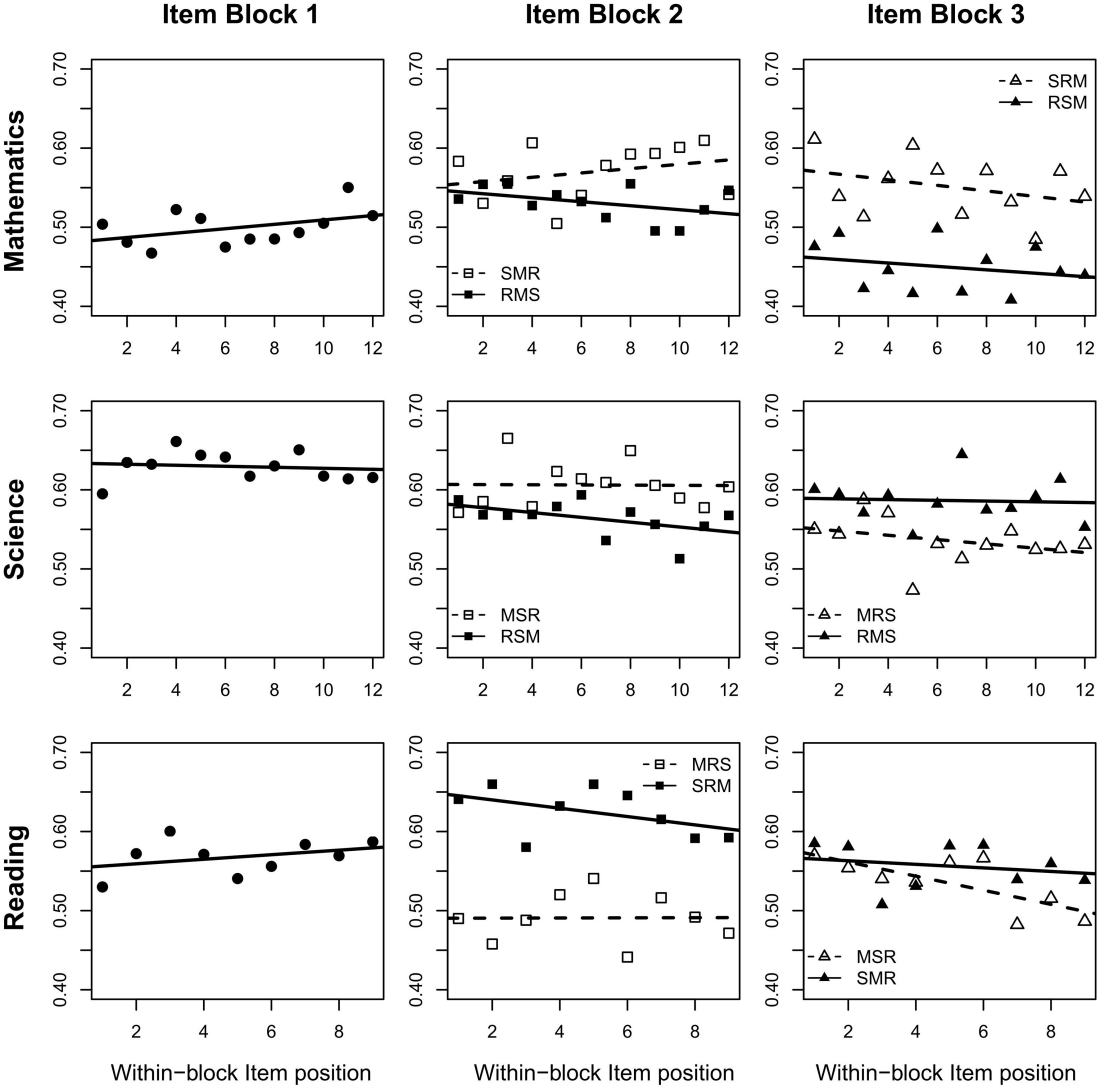
Proportions of correct responses across all items of a domain depending on the item position within the test and the domain order.

The results of our analyses are best illustrated by considering the item means, depending on the test form, the block position, and the within-block item position (Figure 2). Mathematics items were, on average, answered correctly more often when presented in the middle of the test instead of at the beginning. However, this effect differs across domain-orders and was stronger when science items preceded mathematics items (*S-M-R*; ${\hat{\gamma }}_{M2}^{\left(S\right)}$ = 0.415, *SE* = 0.085, *p* < 0.001). Compared to this result, the logits were significantly lower (*R-M-S*; ${\hat{\gamma }}_{M2}^{\left(\times \right)}$ = −0.266, *SE* = 0.088, *p* = 0.003) but still significant when reading was administered first (*R-M-S*; ${\hat{\gamma }}_{M2}^{\left(S\right)}+{\hat{\gamma }}_{M2}^{\left(\times \right)}$ = 0.148, *SE* = 0.148, *p* = 0.022). The results of Model M1 implied decreasing probabilities of correct responses to mathematics items presented in the end of the test. However, as Model M2r revealed this finding was mainly driven by the comparably low rates of correct responses to mathematics items of students working on the test order *R-S-M*. Their mean logit was even lower compared to students who answered mathematics in the beginning of the test (${\hat{\gamma }}_{M3}^{\left(SR\right)}+{\hat{\gamma }}_{M3}^{\left(\times \right)}$ = −0.180, *SE* = 0.080, *p* = 0.013). In contrast, logits continued to be higher in the third position, compared to the first block position, when the three domains were presented in the order *S-R-M* (${\hat{\gamma }}_{M3}^{\left(SR\right)}$ = 0.338, *SE* = 0.084, *p* < 0.001). Results for science visualized in Figure 2 provide a quite consistent picture of lower logits in later block positions. Nevertheless, the block position effects were also moderated by the domain order. Logits of science items in block position *b* = 2 following mathematics items were on average significantly smaller (${\hat{\gamma }}_{S2}^{\left(M\right)}$ = −0.196, *SE* = 0.076, *p* = 0.010) and even more negative when science was assessed after working on reading items (${\hat{\gamma }}_{S2}^{\left(\times \right)}$ = -0.268, *SE* = 0.089, *p* = 0.002). The domain order continued to moderate the block position effect in third block position relative to the first (${\hat{\gamma }}_{S3}^{\left(\times \right)}$ = 0.246, *SE* = 0.079, *p* = 0.002).

Block position effects in reading items were most strongly moderated by the domain order. In line with results of Model M1, results of M2r confirmed an average decrease in logits in reading items when presented in the second item block following mathematic items (${\hat{\gamma }}_{R2}^{\left(M\right)}$ = −0.428, *SE* = 0.078, *p* < 0.001). However, M2r also revealed an average increase in logits of reading items at block position two, when science items were presented first (0.295, *SE* = 0.080, *p* < 0.001). When reading was assessed in the third instead of the first block of the test, the mean logit was also significantly lower (${\hat{\gamma }}_{R3}^{\left(MS\right)}$ = −0.246, *SE* = 0.082, *p* = 0.003), but this decline differed not significantly across the domain orders (${\hat{\gamma }}_{R3}^{\left(\times \right)}$ = 0.132, *SE* = 0.102, *p* = 0.197).

Taken together, the empirical results illustrate that multiple item context effects can interact in a complex way. Such interaction effects may remain undetected if analyses focus on just one of several item context effects. This can result in biased item and person parameter estimates and may lead to invalid explanations and interpretations of such effects.

## Discussion

Items are always presented in a context. Differences in item score distributions which depend on the context in which a particular item is presented are denoted as context effects. In recent years, such effects have received more attention suggesting that they may be the rule rather than the exception (Leary and Dorans, 1985; Brennan, 1992). However, most recent studies have focused on one specific kind of item context effects, namely item position effects (Meyers et al., 2009).

The aims followed in the present article were 2-fold. First, we investigated potential interactions between item context effects by considering the effects of the domain order and the item positions simultaneously. Second, we presented the GLMM framework (McCulloch et al., 2008) as a flexible approach for studying multiple context effects. The models proposed in this article were derived considering the peculiarities of the booklet design underlying our data. Therefore, its suitability in other applications must be carefully checked. However, we demonstrated how a (multidimensional) IRT model can be derived that takes several interacting and interdepend item context effects into account. The detailed model derivations may serve as guiding examples for other applications. In general, we followed the basic idea of explanatory IRT models and specified GLMMs with the item context factors as additional covariates.

The main result of the empirical analyses in this study is that two context effects, namely the item position and the domain order effects, may interact substantially. In many achievement tests, it was found that items showed a tendency to become more difficult when presented in later positions of the test. At first glance, this finding was also confirmed in our analyses, when we exclusively focused on item position effects. However, including the domain order as an additional item context factor revealed a much more complex pattern. Items can become easier as well as more difficult depending on the domains presented in the beginning of the test. These results are also theoretically challenging, as they are hardly consistent with widely accepted explanations of item position effects as fatigue or practice effects. In fact, in some cases, domain order effects appeared to be much stronger than position effects. For example, the difference in the mean logits of reading items presented in the second item block between tests of the domain order *S-R-M* and *M-R-S* was 0.723. As the standard deviation of the latent reading proficiency was *s*(θ_*R*_) = 0.77, this effect corresponds to a standardized effect of Cohen's *d* = 0.940.

Note that these logit differences between groups of test takers with different versions of test are only interpretable as domain order effects because of the randomized assignment of the test forms. In nonrandomized test designs (e.g., with self-selected test versions) the same logit differences could also reflect true mean differences in the distributions of the latent variables (i.e., the person parameters) between groups with different domain order preferences. In practical applications of complex test and item designs, the analyses of item context effects should be part of the quality assurance, just like analyses of differential item functioning (DIF), item DRIFT or other approaches to check model assumptions. Our findings suggest that reliable analyses of item context effects require (a) strong test and items designs, including randomized assignment of test forms, and (b) to take potential dependencies and interactions between multiple item context effects into account by analyzing them simultaneously.

Despite the substantial item context effects, the distributions of random item and random person effects are very similar across the models M0, M1, M2, and M2r. The estimated variances of item and person parameters as well as the estimated correlation structures of the three domain-specific latent proficiencies hardly differ across models (see Table 5). Considering the substantial item context effects found in our data example, with effect sizes close to one, this finding reveals that variances and correlations of random effects can be insensitive to such effects. Hence, stability of estimated variance-covariance structures across models does not imply that item context effects are negligible. Note that these findings are not sufficient to rule out potentially biased correlations with external variables of interest. In general, item context effects induce construct irrelevant variance and may lead to flawed correlation coefficients. This issue has been demonstrated in the case of item position effects (Nagy et al., 2018a,b).

### Practical Implications

Our study clearly indicates that the domain order can substantially affect the response behavior in mixed domain booklet designs in achievement tests. This result is worrisome, as designs of this kind are quite common in many LSAs of student achievement. In these assessments, a lot of effort is made to account for position effects by using booklet designs with balanced block positions. However, in most designs, domain orders are not balanced, and are sometimes even perfectly confounded with block positions. As a consequence, the impact of position effects and domain order effects on test results cannot be separated (Brennan, 1992).

Our results strongly indicate that domain order effects are an issue of concern when assessing student achievement. Careful development of booklet designs would not only enable researchers to quantify the impact of domain orders on individuals' item responses, but also to derive more purified ability estimates. In most cases, item context effects are expected to be a nuisance rather than a benefit. The models used here may not only be used to statistically control for item context effects but to obtain person parameter estimates adjusted for item context effects. GLMMs allow for the computation of the empirical Bayes estimates of individual proficiency levels. Due to the specification of the fixed part of the more complex models M1 and M2 with the first item block as the reference block, all person parameters were estimated as though *all three* domains were administered at the beginning of the test. However, further work is needed to learn more about the benefits and limitations of employing such complex scoring procedures.

Note that taking the alternative route of employing only one fixed order of domains to all subjects in a study is not a solution to the problem. Domain order effects, as well as position effects, are as likely to occur but, in contrast to systematically rotated booklet designs, it is not possible to quantify and control them (Brennan, 1992). This argument also applies to the typical procedure of applying different tests in a fixed order to all individuals participating in a study. A sequence of tests assessing different domains is similar to a sequence of item blocks assessing different domains. Taken together, much more work is needed in order to gain a fuller understanding of the unwarranted side effects of exposing individuals to sequences of domain-specific tests or test parts.

### Limitations and Further Research

As in any other empirical study, this study is affected by limitations which call for further research. The present design does not enable an estimation of a “pure” position effect on the basis of test takers working on only one domain. Although not strictly necessary for examining the moderating role of domain order effects, estimates of “purified” position effects might serve as a useful benchmark for evaluating the size of domain order effects.

Although the modeling approach proposed turned out to be complex, the resulting models might still appear to be overly simplified. For example, the GLMM framework is restricted to one-parameter IRT models. It would be interesting to implement the proposed models in different frameworks, allowing for more complex measurement models, such as the two or three-parameter IRT model. A further point that might be criticized is that our modeling approach did not include random item context effects. Such effects can, in principle, be estimated in the GLMM framework when simpler models are envisaged. However, we did estimate item position models (M1), including random effects on the person and item side. However, as the results indicated that the models were overparameterized, we did not pursue these models any further in this article (results available from the first author).

The results of our study cannot be generalized automatically to other multidimensional tests that are used for assessing different theoretical constructs. The analyses of multiple context effects and interactions between them have never or rarely been tested systematically. This is an area of further research.

This paper was not intended to provide a theoretical explanation of the various item context effects we found empirically: In the existing literature, fatigue effects, practice effects, and backfire effects (Leary and Dorans, 1985; Tourangeau and Rasinski, 1988; Ackerman and Kanter, 2009; Nagy et al., 2018a) are discussed as underlying mechanisms of item context effects. The contemporary considerations of several context effects may support or challenge such interpretations. Given the accumulation of evidence about the impact of context effects on individuals' test behavior, more investigations are needed in which these phenomena are investigated from a substantive perspective to elucidate the underlying psychological mechanisms.

## Author Contributions

NR was responsible for the theoretical treatise on the conceptualization of item context effects and drafted the manuscript. He performed the theory-based derivation of the presented models and conducted all data analyses in R, including the programming of graphs. GN contributed to the preparation of the manuscript and the presentation of the results. He was involved in writing of all parts of the manuscript and was also responsible for funding the research project. AF Planned and initiated the original study as a part of the MakAdapt project, including the funding thereof. He created the item- and test design and coordinated the assessment and the data collection. He was also involved in the preparation of the manuscript for publication. BN and MB contributed to the preparation of the manuscript and the presentation of the results. Both were involved in writing of all parts of the manuscript and were also responsible for funding the research project. All coauthors provided critical revisions. All authors were involved in interpreting the results and approved the final version of the manuscript for submission.

### Conflict of Interest Statement

The authors declare that the research was conducted in the absence of any commercial or financial relationships that could be construed as a potential conflict of interest.

## Footnotes

^1^Existence of random item position effects across items and persons were tested in all three domains by means of LR Tests. Random item position effects were either not significant or perfectly correlated with other random effects in the model. The later indicates overparameterization of the GLMM (Baayen et al., 2008).

^2^Estimated standard deviations and correlations of the random effects obtained by Models M2 and M2r are nearly identical. Therefore, results of Model M2 are not included in Table 5.
