# Supplementary material for: Modeling Multiple Item Context Effects With Generalized Linear Mixed Models
Source: Front Psychol. 2019 Feb 25;10:248. doi: 10.3389/fpsyg.2019.00248 (PMC6397884; doi:10.3389/fpsyg.2019.00248)
Supplement: Supplementary file 1 [file Data_Sheet_1.pdf]

# Appendix to Modeling Multiple Item Context Effects with Generalized Linear Mixed Models

**Norman Rose[1*], Gabriel Nagy[2], Benjamin Nagengast[1], Andreas Frey[3,4], Michael Becker[2,5]**

[1]Hector Research Institute of Education Sciences and Psychology, University of Tübingen, Tübingen, Germany

[2]Leibniz Institute for Science and Mathematics Education, Kiel, Germany

[3]Institute of Psychology, Department of Educational Psychology: Measurement, Evaluation & Counseling, Goethe-University Frankfurt, Frankfurt, Germany

[4]Centre for Educational Measurement (CEMO), Faculty of Education, University of Oslo, Oslo, Norway

[5] German Institute for International Educational Research, Frankfurt a.M./Berlin, Germany

*** Correspondence:**
Norman Rose
norman.rose@uni-tuebingen.de

## Appendix

In this appendix, the model equation of Model M2 (see Equation 8) is derived step-by-step. In general interaction effects can be conceptualized as varying regressions coefficients whose values depend on covariates also denoted by *moderators*. Formally, that means that a regression coefficient of an independent variable $X$ in a regression $E(Y \mid X, \boldsymbol{Z})$ is not a constant but a function $f(\boldsymbol{Z})$ of the potentially multidimensional moderating covariate $\boldsymbol{Z}$. We derive Model M2 using this notation.

To allow for interaction effects between the block position, the domain ($D$) and the domain order ($T$), the coefficient $\alpha_{dbt}$ in Equation 7 was defined as a function $f(D, T)$ of the domain ($D$) and the domain order ($T$). Hence, the effect of the block position on the item response is potentially moderated by the domain of the items and the domain order in the test booklet. Similarly, the coefficient $\lambda_{dbt}$ of the within-block item position was defined as a function $f(D, B, T)$. This means that the effect of the position at which items within an item block can be moderated by the domain ($D$) of the items, the block position ($B$) and the domain order ($T$).

There are two coefficients $\alpha_{d2t}$ and $\alpha_{d3t}$ which can be written as

$$\begin{aligned}\alpha_{d2t} &= \left(\gamma_{M2}^{(S)} + \gamma_{M2}^{(\times)} T_{RMS}\right) I_M + \left(\gamma_{S2}^{(M)} + \gamma_{S2}^{(\times)} T_{RSM}\right) I_S + \left(\gamma_{R2}^{(M)} + \gamma_{R2}^{(\times)} T_{SRM}\right) I_R \\ \alpha_{d3t} &= \left(\gamma_{M3}^{(SR)} + \gamma_{M3}^{(\times)} T_{RSM}\right) I_M + \left(\gamma_{S3}^{(MR)} + \gamma_{S3}^{(\times)} T_{RMS}\right) I_S + \left(\gamma_{R3}^{(MS)} + \gamma_{R3}^{(\times)} T_{SMR}\right) I_R\end{aligned} \quad \text{(A1)}$$

Hence, for each domain administered in the second and third block position, one of the two domain orders was chosen as the reference. For example, if mathematics items were presented in the middle of the test ($b$ = 2), the domain order $S$-$M$-$R$ was arbitrarily chosen as the reference. The average block position effect of mathematics items when presented in the middle of a test with the domain order $S$-

*M-R* was $\gamma_{M2}^{(S)}$, whereas the average block position effect of mathematics items when presented in the middle of a test with the reversed domain order (*R-M-S*) was the sum $\gamma_{M2}^{(R)} = \gamma_{M2}^{(S)} + \gamma_{M2}^{(\times)}$. $\gamma_{M2}^{(\times)}$ is the difference in the block position effects in mathematics items in $b = 2$ between the two domain orders *S-M-R* and *R-M-S*: $\gamma_{M2}^{(\times)} = \gamma_{M2}^{(R)} - \gamma_{M2}^{(S)}$.

The coefficient $\lambda_{dbt}$ refers to the within-block item position effects, that is, the logit change in a randomly chosen item *j* of domain *d* when presented in the last position of block *b* instead of the first position of the same block, in a test with the domain order $T = t$. It is assumed that $\lambda_{dbt}$ varies non-randomly as a function $f(D, T, B)$,that is, domain-specific position effects may be moderated by the block position and the domain order of test booklets. $\lambda_{dbt}$ can be decomposed into simpler functions $f_D(B, T)$ and $f_{Db}(T)$, so that

$$\begin{aligned}\lambda_{dbt} &= f\left(D,B,T\right)\\ &= f_M\left(B,T\right)I_M + f_S\left(B,T\right)I_S + f_R\left(B,T\right)I_R\end{aligned} \quad \text{(A2)}$$

where the functions $f_D(B, T)$ represent the domain-specific position effects depending on the block position ($B$) in which the items of domain $D = d$ were presented and the order of domains $T$ in the test booklet. Each function $f_D(B, T)$ can further be written as

$$f_D\left(B,T\right) = f_{D1}\left(T\right)B_1 + f_{D2}\left(T\right)B_2 + f_{D3}\left(T\right)B_3 \quad \text{(A3)}$$

The functions $f_{Db}(T)$ refer to the position effects in items of domain $D = d$ presented in block $B = b$, which might be moderated by the order of domains $T = t$. The functions $f_{Db}(T)$ can be written as a weighted sum of indicator variables $T_{MSR}$, $T_{MRS}$, $T_{SMR}$, $T_{SRM}$, $T_{RMS}$, and $T_{RSM}$. Note that only two of the indicator variables are required for each function because only two possible domain orders exist for each function $f_{db}(T)$. We obtained six functions $f_{db}(T)$, given by

$$\begin{aligned}
f_{M1}\left(T\right) &= \kappa_{M1}\\
f_{M2}\left(T\right) &= \kappa_{M2}^{(S)}T_{SMR} + \kappa_{M2}^{(R)}T_{RMS}\\
f_{M3}\left(T\right) &= \kappa_{M3}^{(SR)}T_{SRM} + \kappa_{M3}^{(RS)}T_{RSM}\\
f_{S1}\left(T\right) &= \kappa_{S1}\\
f_{S2}\left(T\right) &= \kappa_{S2}^{(M)}T_{MSR} + \kappa_{S2}^{(R)}T_{RSM}\\
f_{S3}\left(T\right) &= \kappa_{S3}^{(MR)}T_{MRS} + \kappa_{S3}^{(RM)}T_{RMS}\\
f_{R1}\left(T\right) &= \kappa_{R1}\\
f_{R2}\left(T\right) &= \kappa_{R2}^{(M)}T_{MRS} + \kappa_{R2}^{(S)}T_{SRM}\\
f_{R3}\left(T\right) &= \kappa_{R3}^{(MS)}T_{MSR} + \kappa_{R3}^{(SM)}T_{SMR}
\end{aligned} \quad \text{(A4)}$$

The letters in the superscripts of the coefficients $\kappa_{db}^{(\cdot)}$ indicate previously presented domains and their order. Three functions $f_{M1}(T)$, $f_{S1}(T)$, and $f_{R1}(T)$ of the domain-specific item position effects in the first item block were reduced to constants $\kappa_{M1}$, $\kappa_{S1}$, and $\kappa_{R1}$. This results from the fact that item position effects cannot be moderated by an unknown order of the following two domains. In order to represent

a possible moderation of the within-block item position effects in block two and three by the domain order, we reparameterized the functions $f_{d2}(T)$ and $f_{d3}(T)$ by choosing reference domain orders, so that

$$\begin{aligned}
f_{M2}\left(T\right) &= \kappa_{M2}^{(S)} + \kappa_{M2}^{(\times)} T_{RMS} \\
f_{M3}\left(T\right) &= \kappa_{M3}^{(SR)} + \kappa_{M3}^{(\times)} T_{RSM} \\
f_{S2}\left(T\right) &= \kappa_{S2}^{(M)} + \kappa_{S2}^{(\times)} T_{RSM} \\
f_{S3}\left(T\right) &= \kappa_{S3}^{(MR)} + \kappa_{S3}^{(\times)} T_{RMS} \\
f_{R2}\left(T\right) &= \kappa_{R2}^{(M)} + \kappa_{R2}^{(\times)} T_{SRM} \\
f_{R3}\left(T\right) &= \kappa_{R3}^{(MS)} + \kappa_{R3}^{(\times)} T_{SMR}
\end{aligned} \tag{A5}$$

Using this model formulation, the parameters $\kappa_{db}^{(\times)}$ represent differences in domain-specific within-block item position effects between test forms with different domain orders, but where the items of domain $D = d$ were always presented in block $B = b$. For example, $\kappa_{M2}^{(\times)}$ is equal to $\kappa_{M2}^{(R)} - \kappa_{M2}^{(S)}$, and represents the difference in average position effects in mathematics items presented in the second item block between test booklets with the domain order *R-M-S* instead of *S-M-R*. Therefore, the parameters $\kappa_{db}^{(\times)}$ refer to the potential four-way interaction effects between the domain, the block position, the within-block item position, and the domain order. Inserting the terms of Equations A4 and A5 into Equation A2 yields the final model equation of $\lambda_{dbt}$ which can finally be written as

$$\begin{aligned}
\lambda_{dbt} = &\left[\kappa_{M1}B_1 + \left(\kappa_{M2}^{(S)} + \kappa_{M2}^{(\times)}T_{RMS}\right)B_2 + \left(\kappa_{M3}^{(SR)} + \kappa_{M3}^{(\times)}T_{RSM}\right)B_3\right]I_M \\
&+ \left[\kappa_{S1}B_1 + \left(\kappa_{S2}^{(M)} + \kappa_{S2}^{(\times)}T_{RSM}\right)B_2 + \left(\kappa_{S3}^{(MR)} + \kappa_{S3}^{(\times)}T_{RMS}\right)B_3\right]I_S \\
&+ \left[\kappa_{R1}B_1 + \left(\kappa_{R2}^{(M)} + \kappa_{R2}^{(\times)}T_{SRM}\right)B_2 + \left(\kappa_{R3}^{(MS)} + \kappa_{R3}^{(\times)}T_{SMR}\right)B_3\right]I_R
\end{aligned} \tag{A6}$$

Multiplying out the brackets of Equations 9 and 14 and inserting the resulting terms into Equation 7 yields:

$$\begin{aligned}
l\left(Y_{ijdpb}\right) = &\beta_{ijM}I_M + \beta_{ijS}I_{jS} + \beta_{ijR}I_R \\
&+\kappa_{M1}B_1I_MX_{pb} + \kappa_{S1}B_1I_SX_{pb} + \kappa_{R1}B_1I_RX_{pb} \\
&+\gamma_{M2}^{(S)}I_MB_2 + \gamma_{M2}^{(\times)}T_{RMS}I_MB_2 + \gamma_{S2}^{(M)}I_SB_2 + \gamma_{S2}^{(\times)}T_{RSM}I_SB_2 + \gamma_{R2}^{(M)}I_RB_2 + \gamma_{R2}^{(\times)}T_{SRM}I_RB_2 \\
&+\gamma_{M3}^{(SR)}I_MB_3 + \gamma_{M3}^{(\times)}T_{RSM}I_MB_3 + \gamma_{S3}^{(MR)}I_SB_3 + \gamma_{S3}^{(\times)}T_{RMS}I_SB_3 + \gamma_{R3}^{(MS)}I_RB_3 + \gamma_{R3}^{(\times)}T_{SMR}I_RB_3 \\
&+\kappa_{M2}^{(S)}B_2I_MX_{pb} + \kappa_{M2}^{(\times)}T_{RMS}B_2I_MX_{pb} + \kappa_{M3}^{(SR)}B_3I_MX_{pb} + \kappa_{M3}^{(\times)}T_{RSM}B_3I_MX_{pb} \\
&+\kappa_{S2}^{(M)}B_2I_SX_{pb} + \kappa_{S2}^{(\times)}T_{RSM}B_2I_SX_{pb} + \kappa_{S3}^{(MR)}B_3I_SX_{pb} + \kappa_{S3}^{(\times)}T_{RMS}B_3I_SX_{pb} \\
&+\kappa_{R2}^{(M)}B_2I_RX_{pb} + \kappa_{R2}^{(\times)}T_{SRM}B_2I_RX_{pb} + \kappa_{R3}^{(MS)}I_RX_{pb} + \kappa_{R3}^{(\times)}T_{SMR}B_3I_RX_{pb}
\end{aligned} \tag{A7}$$

The parameters $\beta_{ijd}$ are random slopes. Inserting the respective model equations of the random slopes (see Equation 3) into Equation A7 yields the final model equation of Model M2 (see Equation 8),

which was fitted by means of the *glmer* function in R. Note that the parameters $\gamma_{M1}$, $\gamma_{S1}$, and $\gamma_{R1}$ in Equation 8 differ in their meaning from $\gamma_M$, $\gamma_S$, and $\gamma_R$ in models M0 and M1. In Model M2 these parameters are the average logits across items and persons in the respective domain in the first item block, which was chosen as the reference.
